# East London Experience with Enteric Fever 2007-2012

**DOI:** 10.1371/journal.pone.0120926

**Published:** 2015-03-19

**Authors:** Jayshree Dave, Michael Millar, Horst Maxeiner, Joanne Freedman, Rachel Meade, Caryn Rosmarin, Matthew Jordan, Nick Andrews, Richard Holliman, Armine Sefton

**Affiliations:** 1 Public Health Laboratory London, Public Health England, London, United Kingdom; 2 Division of Infection, Royal London Hospital, Barts Health NHS Trust, London, United Kingdom; 3 Travel and Migrant Health Section, Centre for Infectious Disease Surveillance and Control, Public Health England, London, United Kingdom; 4 Statistics, Modelling and Economics Department, Centre for Infectious Disease Surveillance and Control, Public Health England, London, United Kingdom; 5 Centre of Immunology and Infectious Disease, Blizard Institute, Barts and The London School of Medicine & Dentistry, Queen Mary University of London, London, United Kingdom; Cardiff University, UNITED KINGDOM

## Abstract

**Purpose:**

The clinical presentation and epidemiology for patients with enteric fever at two hospitals in East London during 2007–2012 is described with the aim to identify preventive opportunities and to reduce the cost of treatment.

**Methods:**

A retrospective analysis of case notes from patients admitted with enteric fever during 2007 to 2012 with a microbiologically confirmed diagnosis was undertaken. Details on clinical presentation, travel history, demographic data, laboratory parameters, treatment, patient outcome and vaccination status were collected.

**Results:**

Clinical case notes were available for 98/129 (76%) patients including 69 *Salmonella enterica* serovar Typhi (*S*. Typhi) and 29 *Salmonella enterica* serovar Paratyphi (S. Paratyphi). Thirty-four patients (35%) were discharged from emergency medicine without a diagnosis of enteric fever and then readmitted after positive blood cultures. Seventy-one of the 98 patients (72%) were UK residents who had travelled abroad, 23 (23%) were foreign visitors/new entrants to the UK and four (4%) had not travelled abroad. Enteric fever was not considered in the initial differential diagnosis for 48/98 (49%) cases. The median length of hospital stay was 7 days (range 0–57 days). The total cost of bed days for managing enteric fever was £454,000 in the two hospitals (mean £75,666/year). Median time to clinical resolution was five days (range 1–20). Seven of 98 (7%) patients were readmitted with relapsed or continued infection. Six of the 71 (8%) patients had received typhoid vaccination, 34 (48%) patients had not received vaccination, and for 31 cases (44%) vaccination status was unknown.

**Conclusions:**

Further interventions regarding education and vaccination of travellers and recognition of the condition by emergency medicine clinicians in travellers to South Asia is required.

## Introduction

Enteric fever, often described in the literature as typhoid fever, is characterised by a non-specific illness with low grade fever, malaise, dry cough, and abdominal pain. The infection is caused by *Salmonella enterica* serovar Typhi (*S*. Typhi), and *Salmonella enteric* serovar Paratyphi (*S*. Paratyphi) A, B, or C and is acquired by ingestion of contaminated food and water [1]. Early recognition and public health interventions for enteric fever are important for high risk cases such as those working within the food industry or attending nursery to prevent onward transmission.

It is estimated that globally there are 27 million cases of enteric fever occurring each year [2] with estimates of mortality varying from 216,000 to 600,000 per annum [3, 4]. The majority of cases of enteric fever are reported in South Asia [3]. A recent population based surveillance study indicated a high incidence of enteric fever in India and Pakistan, intermediate incidence in Indonesia, and low incidence in China and Vietnam [5]. Outbreaks of enteric fever in sub-Saharan Africa are increasingly being described, and the incidence appears to be on the decline in Latin America [2, 3, 6].

Lack of access to safe food and water, poor sanitation and a limited public health infrastructure to support health education and vaccination programmes contribute to the on-going risk of enteric fever in both residents of and travellers to endemic countries.

In 2012, 354 laboratory confirmed cases of enteric fever were reported in England, Wales and Northern Ireland (EWNI) by Public Health England of which 303 (86%) were travel related [7]. *S*. Typhi accounted for 50% of cases and 50% were *S*. Paratyphi. Of the total, 303 cases were UK residents who had travelled abroad, the majority were of Indian, Pakistani or Bangladeshi ethnicity who had travelled to South Asia to visit friends and relatives; these findings were consistent with previous years [7].

London accounts for around 40% of cases reported in EWNI [7, 8], with North and East London (particularly the East London Boroughs of Newham and Tower Hamlets) reporting the highest incidence rates [8]. This is most likely due to the high proportion of Indian, Pakistani and Bangladeshi ethnic groups residing in these areas [9]. Those of Bangladeshi ethnicity (most from Sylhet) [10] are particularly concentrated in Tower Hamlets (representing 32% of this population in 2011) [9]. The epidemiology and antibiotic resistance of enteric fever in East London during the period 2005–2010 has been described in an earlier study [8], where the costs to the NHS associated with treating these infections in East London were estimated to be £272,747 over the six years. Following this study, local public health intervention to improve vaccine uptake was undertaken.

The aim of this study was 1) to describe in detail the clinical features, laboratory markers, epidemiology and management of patients with enteric fever presenting at the Royal London Hospital and Newham General Hospital (now part of Barts Health NHS Trust) in East London between 2007 and 2012 and 2) to identify preventive opportunities, and thereby reducing the cost of treating enteric fever in East London.

## Methods

### Inclusion criteria

All patients seen in the emergency medicine department at Newham General Hospital and the Royal London Hospital with blood cultures positive for *S*. Typhi or *S*. Paratyphi during the period 1 January 2007 to 31 December 2012 inclusive were included in this study.

### Data collection and microbiology methods

Microbiological records were reviewed for patients admitted with enteric fever at the Royal London and Newham General hospitals over the study period. This study was designed as a non-interventional, retrospective analysis of case notes from patients admitted with enteric fever at two London hospitals. The clinical case notes of 98/129 (76%) patients admitted during January 2007 to December 2012 with a microbiologically confirmed diagnosis of enteric fever were available for review. We actively searched the case notes for details on clinical presentation, travel history, demographic data, laboratory parameters, treatment and patient outcome including relapse and eradication. *S*. Typhi and *S*. Paratyphi systemic infection was defined by at least one positive blood culture. The organisms were cultured on selective media and identified by API 20E (BioMerieux) or MALDI-TOF MS (Bruker Corporation) and serology. Data from Public Health England enteric fever enhanced surveillance [1, 11] were used to determine the vaccination status and travel history in each case. Clinical correlates with isolate antibiotic susceptibilities will be the subject of a further report.

### Diagnosis and patient management

Patients with suspected enteric fever were admitted via emergency medicine and treated empirically with ceftriaxone, azithromycin or ciprofloxacin; therapy was changed as necessary after antimicrobial susceptibility results became available. Clinical advice was provided by medical staff in the Division of Infection, at Barts Health NHS Trust.

Clinical failure was defined as either persistence or recurrence of presenting symptoms or increase in severity of at least one sign or symptom after seven days of antibiotic treatment for infection. Relapse was defined as recurrence of enteric fever related symptoms confirmed by positive blood culture within one month of initial presentation. Fever clearance time was defined as time from onset of treatment to first recorded temperature of ≤37.5C which persisted for 48 hours or more. Severe infection was defined as multi-organ involvement with sepsis and/or admission to an intensive care unit. Length of hospital stay was defined as the time in days from hospital admission until discharge. Convalescent faecal carriage was defined as a positive faecal culture detected at any time after the end of treatment up to one year following the date of diagnosis. Abnormal biochemistry and haematology results were defined as any values which occurred outside the normal range for the testing laboratory. Biochemistry data collected included alkaline phosphatase (ALP), alanine transaminase (ALT), bilirubin, and C-reactive protein (CRP) values. Haematology data included white cell count (WCC), neutrophils, lymphocytes, haemoglobin, and platelets for each patient.

### Data analysis

An Excel database was created to manage patient information. Patient details were anonymised and indexed using a study reference number and age in years. Comparisons between *S*. Typhi and *S*. Paratyphi were undertaken using the Kruskal-Wallis test (for continuous data such as length of stay) or Fisher’s exact test (for proportions). A 5% significance level was used.

### Ethics statement

This study involves secondary use of non-identifiable patient information, previously collected in the course of normal care, anonymised and de-identified prior to analysis. Barts Health NHS Trust and Queen Mary University of London’s Joint Research Management Office confirmed that formal review and approval by an ethics committee (NHS REC review) was not required.

## Results

### Microbiology

During 2007 to 2012, 111 blood cultures positive for *S*. Typhi were identified from 89 patients and 48 blood cultures positive for *S*. Paratyphi were identified from 38 patients.

### Clinical diagnosis

Of the 129 patients with microbiologically-confirmed enteric fever during the study period, clinical case notes were available for 98/129 (76%) patients; including 69 patients with *S*. Typhi infection and 29 patients with *S*. Paratyphi infection. All blood cultures became positive within 48 hours of sampling following admission of patients. Thirty-four patients (35%) were initially discharged from emergency medicine without a clinical diagnosis of enteric fever and only subsequently readmitted when blood cultures became positive. Stool cultures were available for 43 of the 98 patients during hospital stay and 19 of these were positive (15 *S*. Typhi and four *S*. Paratyphi).

### Demographics

Of the 98 patients reviewed, 18 (18%) were aged 17 years and under of which seven (39%) were under five years; 28 (29%) were aged 15–24 years and 44 (45%) were aged 25–39 years (Table 1). The median age was 25.5 years [range 0.5–68 years]. Infection was more common in males (51/98, 52%) compared to females (47/98, 48%) but this was not statistically significant. Forty one patients (42%) were Bangladeshi, 31(32%) were Indian and 18 (18%) were Pakistani.

**Table 1:** Demographics and enhanced surveillance.

	n	%
Males	51	52
Females	47	48
*Age (years)*		
*< 5 years old*	7	7.1
0–14	15	15.3
< 18 years old	18	18.4
15–24	28	28.6
25–39	44	44.9
40–59	8	8.2
60+	3	3.1
*Ethnicity*		
Bangladeshi	41	41.8
Pakistani	18	18.4
Indian	31	31.6
Black	4	4.1
Caucasian	2	2
Other ethnicity	2	2
*Travel abroad/*	71	72.4
*Country visited*		
Bangladesh	28	39.4
Bangladesh / Dubai	1	1.4
Bangladesh / India	2	2.8
Ghana	1	1.4
India	18	25.4
India / USA	1	1.4
India / Thailand	2	2.8
India / Kuwait	1	1.4
India / Pakistan	1	1.4
Pakistan	13	18.3
Turkey	1	1.4
Nepal	1	1.4
Nigeria	1	1.4
Visitor to the UK / new entrants	23	23.5
No history of foreign travel	4	4.1
Vaccination <3 years prior to travel*		
Yes	6	8.5
No	34	47.9
Not Known	31	43.7

* Excludes new entrants & visitors

### Travel history

Of the total, 71/98 (72%) patients were UK residents who had travelled abroad, 23 (23%) were foreign visitors or new entrants to the UK and four (4%) had not travelled abroad. Of UK residents who had travelled abroad, 58 (82%) had visited friends and relatives; 29 (50%) had visited them in Bangladesh, 20 (34%) in India, and 12 (21%) in Pakistan. Two patients had visited both Bangladesh and India and one visited both India and Pakistan. The remaining 13 patients travelled for a holiday to other countries (Table 1). Where duration of travel information was available (N = 62), the median duration of travel was 31 days [range 3–343 days].

### Clinical features

The predominant presenting symptoms are described in Table 2. Although blood cultures were taken in the context of fever in returning travellers, enteric fever was not initially documented as part of the differential diagnosis in 48 (49%) of the 98 cases. Clinical symptoms, excluding pyrexia prior to admission, were documented in 95 patients with a median of 5 days [range 1–49 days] between onset and admission.

**Table 2:** Clinical symptoms and signs on admission for 95 patients.

Clinical features	Number of patients (%)
Headache	49 (52)
Diarrhoea	48 (51)
Vomiting	46 (48)
Abdominal pain	45 (47)
Cough	34 (36)
Fever	31 (33)
Loss of appetite	15 (16)
Malaise/lethargy/fatigue	10 (11)
Rigors	8 (8)
Sore throat	6 (6)
Constipation	5 (5)
Myalgia	4 (4)
Hepatosplenomegaly	4 (4)
Night sweats	3 (3)
CNS involvement	2 (2)
Small intestinal perforation	1 (1)

Around half of patients presented with abdominal pain (47%, 45/95), vomiting (48%, 46/95) diarrhoea (51%, 48/95), or headache (52%, 49/95). Fever was reported in 31/95 (33%) and cough in 34/95 (36%). Malaise, fatigue or lethargy were reported in 10/95 (11%) of the patients. Five patients presented with constipation and four patients had hepato-splenomegaly. Eight percent (7/89) of patients were found to be apyrexial on admission but blood cultures were subsequently positive for enteric fever.

There were seven patients with severe symptoms; two with *S*. Paratyphi and five with *S*. Typhi. One pregnant patient developed necrosis of the gall bladder and peritonitis and was admitted to the intensive care unit. One patient had perforation of the small intestine and two patients presented with neurological signs and symptoms. One patient with HIV was admitted to intensive care with septic shock, an ex-premature baby was admitted to special care baby unit and one patient developed sepsis with disseminated intravascular coagulation. Four patients, with fever, abdominal pain and non-specific symptoms had already received treatment following a diagnosis of enteric fever in South Asia. There were no recorded deaths.

For the 93 patients where data was available, the median time prior to hospital admission from date of entry or return to the UK was 15 days [range 1–74] (Table 3). There was no relationship between age and length of time prior to presentation following entry to UK (correlation *r* = 0.12, p = 0.25). This time period also did not differ between *S*. Typhi and *S*. Paratyphi (p = 0.45, Table 3).

**Table 3:** Temporal findings.

	Total Patients n = 98			*S*. Typhi n = 69			*S*. Paratyphi n = 29		
	n	Median	Range	n	Median	Range	n	Median	Range
Length of Hospital Stay (days)	98	7	0–57	69	8	0–57	29	6	0–16
Temperature resolution* (days)	80	5	0–20	53	6	1–20	27	5	0–14
Clinical resolution (days)	81	5	1–20	55	5	1–20	26	4	1–14
Duration to admission from entry to the UK (days)	93	15	1–74	65	14	1–74	28	17	5–56

* does not include 11 patients who were discharged with pyrexia

The time to admission between UK residents and visitors was not significantly different (p = 0.388, adjusted for ties). The UK residents’ median time to admission was 16 days and the visitors’ median time to admission was 13 days.

### Length of hospital stay

The median length of hospital stay was 7 days [range 0–57 days] for the 98 patients studied. The total number of hospital bed days allocated was 908 for the 105 patient episodes. Based on local calculations for hospital admission costs of £500 per day, the total cost of bed days for enteric fever was £454,000 in the two hospitals over six years (mean £75,666/year). *S*. Typhi infected cases had a median of two more days in hospital than *S*. Paratyphi [range 0–57 (*S*. Typhi), 0–16 (*S*. Paratyphi)], although this difference was not significant (p = 0.11, Table 3).

### Temperature and clinical resolution

The median time to resolution of pyrexia in 80 patients was 5 days [range 0–20] (Table 3). Among the eleven patients who had an unresolved temperature prior to discharge, there were four patients aged 1–14 years, three patients aged 15–24 years, three patients aged 25–39 years and one patient aged 40–59 years. Eight of these patients were sent home on oral antibiotics, one of whom was a foreign national who continued treatment in the Netherlands. Two children were seen daily at the hospital and given outpatient intravenous antibiotic therapy.

One of these 11 patients with unresolved pyrexia was seen in A&E but not admitted, and did not receive antibiotic treatment, as this patient was lost to follow-up despite several attempts by the emergency medicine staff to contact the patient. For the remaining 10 patients: seven received ceftriaxone (including one in combination with gentamicin and clarithromycin), one received co-amoxiclav, one received cephalexin, and one received ciprofloxacin during their hospital stay. The range of hospital stay for these 11 patients was 0–14 days (Table 3). The reasons for early discharge were not documented.

For the 81 patients where data was available, median time to clinical resolution following the start of treatment was five days [range 1–20] (Table 3). Clinical resolution was significantly longer in patients with *S*. Typhi (p = 0.04) compared to those infected with *S*. Paratyphi. Similarly, temperature resolution was prolonged in patients with *S*. Typhi (p = 0.01) compared to those with *S*. Paratyphi.

Twenty-one patients (21%) had clinical failure based on the study definition. The median clinical resolution for these patients was 10 days [range 8–20 days]. Of these patients, two had concurrent cryptosporidium infection and three had severe, complicated enteric fevers: one pregnant patient was septic with peritonitis following gall bladder necrosis; one had intestinal perforation and one patient had severe sepsis with disseminated intravascular coagulation. The remaining 16 patients with clinical failure had a median clinical resolution of 10 days [range 8–16 days]. Reasons for this variation were unclear.

In three of the four patients who did not receive antibiotics enteric fever was not considered in the differential diagnosis and all four were lost to follow up despite attempts by emergency medicine staff to contact these patients. Two of these patients were visitors to the UK and may have returned home.

### Recurrence of Infection

Seven of 98 (7%) patients who had previously received treatment for enteric fever, were readmitted with further positive blood cultures for *S*. Typhi or *S*. Paratyphi. Six of the seven patients had previously received at least seven days of appropriate treatment, and one patient received a total of five days treatment (Table 4). These seven patients were treated with ceftriaxone empirically. Six of these patients had *S*. Typhi infection and one had *S*. Paratyphi. Two patients were aged 0–14 years, one aged 15–24 years, three aged 25–39 years and one patient was over 60 years old.

**Table 4:** Patients readmitted with infection.

Age	Species	Antibiotic Therapy Admission 1	Duration (days)	Discharge Antibiotic Therapy	Days to relapse	Antibiotic Therapy Admission 2	Duration (days)	Discharge Antibiotic Therapy
5	*S*. Typhi	Ceftriaxone	13	Ceftriaxone	21	Cefriaxone	19	
						Amikacin	13	
11	*S*. Typhi	Ceftriaxone	6	Ceftriaxone	47	Ceftriaxone	8	Ciprofloxacin
		Clarithromycin	1			Clarithromycin	-	
23	*S*. Paratyphi	Ceftriaxone	2	Azithromycin	2	Ceftriaxone	13	
						Azithromycin	2	
28	*S*. Typhi	Amoxicillin & Gentamicin followed by Ceftriaxone & Amikacin	11		27	Ceftriaxone	14	Ceftriaxone
35	*S*. Typhi	Ceftriaxone	7	Ciprofloxacin	42	Ceftriaxone	1	Amoxicillin
						Ciprofloxacin	6	
						Amoxicillin	3	
38	*S*. Typhi	Ceftriaxone	5		23	Ceftriaxone	2	Azithromycin
		Ciprofloxacin	5					
66	*S*. Typhi	Ceftriaxone	10		54	Ceftriaxone	14	Ciprofloxacin
		Amoxicillin	7			Metronidazole	14	

### Risk factors

Occupational history was recorded in the medical notes for 31 patients (32%). Nine adult patients (29%) were considered to be at high risk of transmitting infection [1]. Of these seven were food handlers and two worked with children. In addition, there were seven children aged less than five years who were at high risk of transmission.

### Non-travel-associated cases

Four cases were designated as non-travel-associated cases. One of these had travelled to Bangladesh but returned to the UK 55 days before illness onset, therefore not meeting the travel-associated case definition [1]. This case is likely to be a secondary case transmitted from one of two co-travelling family members who both had confirmed typhoid shortly after they returned to the UK. For the remaining three non-travel associated cases, the source of infection was unclear. There were two travel-associated cases that each had co-travelling family members who had also been infected with the same organism before their onset of illness; it is therefore possible that these could also be secondary cases but not enough information was available to determine this.

### Travel history, vaccination status and clinical features

A record of enquiry about malaria prophylaxis was documented in the medical notes for 73/98 (74%) patients, and a malaria screen was performed on 70% (69/98) of patients. However, only 2/98 (2%) patients had a documented record of enquiry about typhoid vaccination.

Table 1 shows the countries visited by patients who were UK residents (N = 71) and their typhoid vaccination status in the three years before travelling abroad. Of the 71 UK residents with enteric fever: six patients (8%) had received typhoid vaccination in the three years before travel, 34 (48%) had not received vaccination and for 31 cases (44%), vaccination status was unknown. Five patients who had received typhoid vaccination were infected with *S*. Typhi and one was infected with *S*. Paratyphi. The age range of these patients was 3 years to 39 years (3, 6, 18, 28, 31, 39). There was no significant difference in time to clinical resolution, resolution of pyrexia or length of hospital stay for the patients who had received the typhoid vaccination compared with those who did not receive vaccination. Seven patients with recurrence of infection had either not received typhoid vaccination or this information was not disclosed.

### Biochemistry

Table 5 provides normal biochemistry ranges. Table 6 shows the biochemistry and haematology findings in patients admitted with enteric fever, while Table 7 indicates the percentage of values outside the laboratory defined normal range measured in the peripheral blood of patients taken on admission and during hospital stay with microbiological proven enteric fever. Individual parameters were available for 73% to 98% of the 98 patients (Table 7). During hospital stay, C-reactive protein (CRP) was elevated (≥5 U/ml) in 87/87 (100%) of patients. Up to 85% of patients had deranged results, during hospital stay, for other parameters: low haemoglobin (60/93, 65%); lymphopaenia (32/92, 35%); thrombocytopaenia (39/93, 42%); raised ALT (66/78, 85%) and elevated ALP (56/81, 69%). Leukocytosis was present in 10/96 (10%) of patients while leukopaenia was observed in 16/96 (17%) of patients.

**Table 5:** Normal biochemistry ranges.

	WCC (x10^9^/L)	Haemaglobin (g/dL)	Neutrophils (x10^9^/L)	Platelets (x10^9^/L)	Lymphocytes (x10^9^/L)
<1	6.0–16.6	11.1–14.1	1.0–7.0	200–550	3.5–11.0
<6	5.0–15.0	11.0–14.0	1.5–8.0	200–490	6.0–9.0
<12	5.0–13.0	11.5–15.5	2.0–8.0	170–450	1.0–5.0
>12	4.0–10.0	-	2.0–7.0	150–410	1.0–3.0
>12 Male	-	13.0–17.0	-	-	-
> 12 Female	-	12.0–15.0	-	-	-

	Alanine Aminotransferase (ALT) U/L	Alkaline Phosphatase (ALP) U/L	Bilirubin μmol/L	C-Reactive Protein (CRP) U/ml	
Adult Male	10–40	30–130	< 21	< 5	
Adult Female	7–35	30–130	< 21	< 5	
0–12 months	13–45	55–310	< 21	< 5	
> 12 months	10–40	-	< 21	< 5	
1 year—10 years	-	55–350	< 21	< 5	
Adolescent	-	< 520	< 21	< 5	

**Table 6:** Biochemistry findings in patients on admission with enteric fever.

	Total patients n = 98			*S*. Typhi Patients n = 69			*S*. Paratyphi Patients n = 29		
	N	Median	Range	n	Median	Range	n	Median	Range
**ALP**	69	165	44–831	49	169	44–831	20	153.5	53–390
**ALT**	63	53	19–256	45	51	19–256	18	64.5	24–226
**Bilirubin**	59	11	2–72	41	12	3–72	18	9	2–33
**WCC**	90	6.3	1.3–16.1	62	6.35	1.3–16.1	28	6.3	2.7–10.6
**Neutrophils**	84	4.2	1.1–14.2	59	4.2	1.1–14.2	25	3.8	1.4–7.2
**Lymphocytes**	85	1.4	0.2–7.0	58	1.4	0.2–7.0	27	1.5	0.6–3.3
**Haemoglobin**									
*Male*	43	14.1	5.5–16.0	33	13.9	9.9–15.3	10	14.5	5.5–16.0
*Female*	42	11.35	8.5–14.9	27	11.2	8.8–14.6	15	11.9	8.5–14.9
**CRP**	63	89	14–297	42	107.5	14–297	21	69	18–140
**Platelets**	86	180	38–339	59	167	38–339	27	208	46–333

**Table 7:** Biochemistry abnormalities at admission and during hospital stay.

		All patients (max N = 98)		*S*. Typhi patients (max N = 69)		*S*. Paratyphi patients (max N = 29)		*S*. Typhi vs *S*. Paratyphi (admission) Fisher's exact test p-value	*S*. Typhi vs *S*. Paratyphi (during stay) Fisher's exact test p-value
		Admission	During stay	Admission	During stay	Admission	During stay		
		n/N (%)	n/N (%)	n/N (%)	n/N (%)	n/N (%)	n/N (%)		
**Haemoglobin** (n = low)		33/85 (38.8)	60/93 (64.5)	24/60 (40.0)	46/65 (70.8)	9/25 (36.0)	14/28 (50.0)	0.81	0.063
**WCC**	Abnormal	11/90 (12.2)	26/96 (27.1)	8/62 (12.9)	22/67 (32.8)	3/28 (10.7)	4/29 (13.8)	1.00	0.079
	High	4/90 (4.4)	10/96 (10.4)	3/62 (4.8)	8/67 (11.9)	1/28 (3.6)	2/29 (6.9)	1.00	0.718
	Low	7/90 (7.8)	16/96 (16.7)	5/62 (8.1)	14/67 (20.9)	2/28 (7.1)	2/29 (6.9)	1.00	0.136
**Neutrophils**		10/84 (11.9)	22/91 (24.2)	7/59 (11.9)	19/64 (29.7)	3/25 (12.0)	3/27 (11.1)	1.00	0.029
**Lymphocytes**	Abnormal	28/85 (32.9)	40/92 (43.5)	22/58 (37.9)	32/64 (50.0)*	6/27 (22.2)	8/28 (28.6)	0.22	0.069
	Low	24/85 (28.2)	32/92 (34.8)	19/58 (32.8)	26/64 (40.6)	5/27 (18.5)	6/28 (21.4)	0.21	0.097
	High	4/85 (4.7)	10/92 (10.9)	3/58 (5.2)	6/64 (9.4)	1/27 (3.7)	4/28 (14.3)	1.00	0.486
**Platelets**	Abnormal	28/86 (32.6)	50/93 (53.8)	23/59 (39.0)	40/65 (61.5)*	5/27 (18.5)	10/28 (35.7)	0.08	0.025
	Low	28/86 (32.6)	39/93 (41.9)	23/59 (39.0)	32/65 (49.2)	5/27 (18.5)	7/28 (25.0)	0.08	0.039
	High	0/86	17/93 (18.3)	0/59	13/65 (20.0)	0/27	4/28 (14.3)		0.575
**Bilirubin** (n = high)		9/59 (15.2)	14/72 (19.4)	8/41 (19.5)	13/51 (25.5)	1/18 (5.6)	1/21 (4.8)	0.25	0.053
**ALP** (n = high)		32/69 (46.4)	56/81 (69.1)	22/49 (44.9)	40/58 (69.0)	10/20 (50)	16/23 (69.6)	0.79	0.788
**ALT** (n = high)		45/63 (71.4)	66/78 (84.6)	32/45 (71.1)	47/56 (83.9)	13/18 (72.2)	19/22 (86.4)	1.00	1
**CRP** (n = high)		63/63 (100)	87/87 (100)	42/42 (100)	60/60 (100)	21/21 (100)	27/27 (100)		

During hospital stay, where data was available (N = 78) ALT was twice or greater than the upper limit of normal in 52 patients (67%). For ALP this was the case for 28% of patients (23/81) and for bilirubin, 8% (6/72).

The percentage of patients with abnormal white blood cell count and neutrophil count was similar at admission for *S*. Typhi and *S*. Paratyphi infections; 13% and 11% respectively (WCC); 12% and 12% respectively (neutrophils), but more patients with *S*. Typhi infection had lymphopaenia (33% vs 19%). There was also a difference in the percentage of patients with thrombocytopaenia on admission (39% *S*. Typhi vs 19% *S*. Paratyphi, p = 0.08).

The most pronounced differences between the two species were observed in lymphopaenia during hospital stay (41% vs 21%, p = 0.097), thrombocytopaenia (49% vs 25%, p = 0.039), and bilirubin (26% vs 5%, p = 0.053). Neutrophils (p = 0.029), platelets (p = 0.025) and bilirubin (p = 0.053) abnormalities were significantly greater in *S*. Typhi compared to *S*. Paratyphi.

## Discussion

This study comprised a retrospective review of enteric fever cases admitted to two hospitals in East London over a seven year period.

The majority of patients reviewed in this study had acquired enteric fever while travelling to visit friends and relatives in India, Pakistan and Bangladesh, with the highest proportion associated with travel to Bangladesh. The East London Borough of Tower Hamlets has a large Bangladeshi resident population, who regularly visit their families in Bangladesh.

Our findings are similar to other published reports [11, 12], and a previous study in East London [8], where around 90% of cases of enteric fever had travelled overseas and most were VFR travellers. It is not possible to estimate the infection rate among overseas travellers from East London as travel statistics are not available at Borough level; however, a report from 2006–7 estimated that the rate of enteric fever associated with visits to India, Pakistan and Bangladesh in VFR travellers from England, Wales and Northern Ireland was almost seven times higher than the rate in non-VFR travellers [13]. Around 20% of visits abroad by UK residents (just under 12 million in 2012) are to visit friends and relatives, and of those, around 7% visit India, Pakistan and Bangladesh [14].

Our findings show that some patients with enteric fever, when seen in a UK emergency medicine department, may either be at risk of not receiving appropriate initial antibiotic treatment (as typhoid has not been considered in the differential diagnosis) or not being followed up for enhanced surveillance (because they cannot be contacted after the blood culture results show they have enteric fever become available). Enteric fever, unlike malaria, seems often not to be considered as a possible diagnosis in returning travellers as the symptoms are often non-specific.

The morbidity and mortality for enteric fever was broadly similar in our patients to that described in the literature [15, 16]. Patients mostly presented with fever and abdominal pain, but non-specific symptoms and respiratory symptoms were also described. Diarrhoea was commonly seen in adults and five percent of patients presented with constipation. Rose spots were not described in our patients, perhaps as they are difficult to detect in an ethnic population with darker skin. Complications were rare in our case series and all patients survived. Interestingly, up to 8% (7/89) of the patients did not have an elevated temperature recorded at presentation. However, these patients received appropriate treatment and this issue highlights the necessity for taking blood cultures in patients with a history of fever and return from an endemic area. In emergency medicine, blood cultures were taken as part of the initial assessment but some patients may have been sent home without relevant investigations because of the non-specific nature of presentation of enteric fever, suggesting under-diagnosis.

Clinical and temperature resolution was documented for 82% of the patients as some patients were discharged when clinically improved, despite an elevated temperature on antibiotic treatment. However, for both features, the median resolution was rapid at five days, which is consistent with the literature [17]. We defined clinical failure as treatment given for more than seven days and for the great majority of the patients clinical resolution was within seven days. For 21% of the patients, it took more than seven days for clinical resolution (median 10 days) and some of these patients had a severe infection or had other concurrent infections. A small number of patients had recurrent infection despite appropriate treatment with ceftriaxone, consistent with previous reports of up to 20% recurrence rates [15–17]. There was no significant difference in length of hospital stay between patients with *S*. Typhi and *S*. Paratyphi as treatment was standardised and outpatient antibiotic treatment was not available locally during the study period. However, we did find that clinical resolution and temperature resolution were significantly prolonged in patients with *S*. Typhi compared to *S*. Paratyphi, despite the relatively small numbers studied. The severity of infection in patients was not different between the two species. The majority of our patients had abnormal laboratory parameters with anaemia, raised CRP and liver transaminases, as previously described in the literature [15, 16, 18]. We found that patients with *S*. Typhi had significantly raised bilirubin, deranged neutrophils and also thrombocytopaenia compared with patients with *S*. Paratyphi. Recent reports have suggested that *S*. Paratyphi has a more severe or a similar presentation to *S*. Typhi [19, 20], however our findings do not support this.

While the median duration of symptoms prior to admission was five days, the range was wide (1–49 days), although in some cases this was difficult to ascertain accurately. The late presentation of some patients with enteric fever in emergency medicine after entry to the UK may reflect poor knowledge of access to health care, cultural and behavioural differences or partial treatment with antibiotics before presentation. Additionally, it is possible that individuals from endemic areas may have developed partial immunity to infection and hence may present later with symptoms. There was also no relationship between late presentation and the species of salmonella (*S*. Typhi or *S*. Paratyphi) causing the infection. Documentation was poor with respect to risk factors for recurrent disease such as co-morbidities. Documentation was poor with respect to risk factors for recurrent disease such as co-morbidities.

Our findings do not fully support the suggestion of others, that there may be a reservoir of asymptomatic individuals in London who have acquired *S*. Typhi or *S*. Paratyphi abroad and who may be a potential source of transmission to other individuals [10]. In a recent report of enteric fever in North London [10], 19 of 329 patients (6%) had infection acquired in the UK, suggesting that there may be a transmission risk in the community. However in our case series, only three individuals (3%) had no relevant exposure history and were therefore possibly acquired in the UK.

There were limitations with this study, the main one being that the clinical record keeping in the hospital case notes (including temperature charts) was found to be variable among different clinicians and notes were only obtained for 76% of patients with a positive blood culture, which is reported to have a sensitivity as low as 40% [21]. Certain data fields collected through enhanced enteric fever surveillance were better completed than others; in particular, information on vaccine history was only available for 40/71 (56%) of patients. There is also a lack of national guidance for treatment for enteric fever, which may have affected how patients were treated on presentation by different clinicians. Finally, despite rigorous attempts by emergency medicine to contact patients, 4% of the patients were lost to follow up.

A very small proportion of patients reviewed were up-to-date with typhoid vaccination before travel. This may be a reflection of the ethnic mix of the population in this study as those of Indian, Pakistani and Bangladeshi ethnicity are less likely to be vaccinated than other ethnicities, particularly if born abroad [12]. More work is required to encourage prospective travellers to seek vaccination before travelling to India, Pakistan or Bangladesh, regardless of ethnicity.

In the UK there are currently two types of vaccine available against *S*. Typhi, a polysaccharide vaccine and an oral, live, attenuated vaccine, for both of which periodic re-immunisation for continued efficacy is recommended [22]; there are currently no licensed vaccines against *S*. Paratyphi. Existing vaccines [23, 24] are not fully protective against *S*. Typhi and have no protection against *S*. Paratyphi and some patients developed infection despite having received vaccination in the previous three years. Travellers, therefore, must also be provided with appropriate health education regarding food and water hygiene precautions prior to departure. Public Health England provides travel advice in four south Asian languages [25]. In 2012, there was a national shortage in the availability of Vi-polysaccharide vaccines [23]; however despite this, there was no increase in typhoid cases reported in the UK [7].

The average current cost per day for hospital admission is 500 GBPs based on local calculations. Using a conservative estimate, enteric fever cost the NHS £454,000 in two East London Hospitals over the six year period in this study. If this was extrapolated to 354 cases of enteric fever annually [7] with a mean stay of nine days, the costs to the NHS for England and Wales could be over 1.5 million GBPs annually. These calculations do not include visits to primary care, emergency medicine, additional time off work and community costs, including public health costs. It is estimated that the incidence of enteric fever is 4–5 times higher in North East London [10] than in the rest of England and these local costs might be reduced by the implementation of focused health education and vaccination for the prevention of enteric fever.

A study in 1994 [26] demonstrated that giving typhoid vaccine to travellers was not cost effective as the incidence of enteric fever was low (0.02%), however, there is evidence to show that in the 21^st^ century, travellers visiting friends and family have an increased incidence of enteric fever [12] and the study in east London [8] showed it would be cost effective to vaccinate travellers. Uptake of typhoid vaccine was poor in our population at the time of the study; we recommend that increasing awareness of the need for typhoid vaccination for travellers should be more focussed in parts of the UK where there is a large resident population of migrants from India, Pakistan and Bangladesh, including second and subsequent generation migrants. Further work is needed to ascertain an accurate picture of uptake of typhoid vaccine across the UK. Measuring the uptake of other vaccines and prophylaxis such as for viral hepatitis, rabies and malaria in the South Asian population may also act as indicators for poor uptake of typhoid vaccine and deserves discussion. One reason for poor uptake might be that cost of vaccines at a travel clinic may be prohibitive, particularly for a family with children, and further work on cultural and behavioural reasons for poor vaccine uptake would be beneficial, informing future improvement plans. In some general practices, patients have to pay prescription costs for typhoid vaccine, while hepatitis A vaccine is free. Commercial travel clinics may charge 50 GBPs for typhoid vaccine, which represents a considerable financial burden for a family with a number of travellers. New fourth generation vaccines are currently in development including conjugate vaccines using various protein carriers and may be active against either *S*. Typhi and *S*. Paratyphi, or both [24].

## Conclusions

Enteric fever is primarily a problem among returning travellers, visitors and new entrants to the UK from South Asia. Recognition of this condition by emergency medicine was suboptimal; 49% of the cases did not have enteric fever documented within the initial differential diagnosis and 35% of patients with enteric fever were sent home from A&E and only admitted to hospital once blood cultures became positive. There was no difference in the severity of infection caused by the two species. However, *S*. Typhi patients had significantly higher bilirubin levels, deranged neutrophils, and thrombocytopaenia. Clinical relapse after a full course of treatment requiring readmission was not uncommon. Preventative measures could be improved and may be cost effective in parts of the UK where there is large resident population from South Asia. Only 8% of the UK residents with enteric fever in our study had definitely received vaccination in the previous three years, 48% of patients had not received vaccination and for 44% of cases vaccination status was unknown. We found a higher percentage of enteric fever due to *S*. Typhi than elsewhere, possibly because of the high proportion of Bangladeshis in the community or low local vaccination rates.

The results of this study indicate that further action is required regarding education and vaccination of travellers to South Asia, and the need for clinicians in emergency medicine to consider diagnostic testing for enteric fever in travellers returning from typhoid endemic areas with a history of fever.
